# Typhlitis in a post-chemotherapy lymphoma patient; images in clinical medicine

**DOI:** 10.3332/ecancer.2010.193

**Published:** 2010-11-11

**Authors:** S Rizzo, M Bellomi

**Affiliations:** 1 Department of Radiology, European Institute of Oncology, via Ripamonti 435, Milan, Italy; 2 University of Milan, Milan, Italy

## Abstract

Typhlitis, also known as neutropenic enterocolitis, occurs primarily in severely immunosuppressed patients undergoing chemotherapy for haemathological and solid tumors. We report a case of a 57 years-old patient who presented typhlitis as a complication during chemotherapy.

A 57-year-old Caucasian male with a history of diffuse large B-cell non-Hodgkin’s lymphoma (NHL), who underwent many different cycles of immunochemotherapy during the period May–December 2009, was referred to our Institution for asthenia, abdominal pain and fever. He had remarkable haematological examinations: WC 0.01x10 ^3^, HB 7.0, PLT 11000, CRP 49 mg/l, ESR 105, LDH 488 UI/l.

A bone marrow biopsy indicated relapse of NHL and a computed tomography (CT) scan of chest-abdomen and pelvis showed enlarged mediastinal and retroperitoneal lymph nodes, an enlarged spleen and a massive hypodense mural thickening of the caecum (Figure 1) and ascending colon (Figure 2).

Typhlitis, a transmural inflammation of the caecum, often with involvement of the ascending colon and ileum, was suspected. A faecal culture was negative for *C. difficile.* The patient was treated with antibiotics, fluids and steroids, resulting in a regression of phlogosis indices in two weeks. Due to fever persistence, interpreted as a sign of disease progression, the patient was thereafter given a further cycle of chemotherapy.

Typhlitis, also known as neutropenic enterocolitis, occurs primarily in severely immunosuppressed patients undergoing chemotherapy for haematological [1] and solid tumours, including breast, lung, colorectal and ovarian cancer [2,3]. However, individuals at risk of developing typhlitis extend beyond oncologic patients. The underlying problem in all of these patients is the existence of absolute neutropenia. Early recognition of typhlitis is paramount to a potentially good outcome as the mortality rate for typhlitis can be as high as 40%–50%, mostly because it is frequently associated with bowel perforation, if left undiagnosed.

Diagnosis is based on imaging and clinical findings, after excluding other pathologies. Neutropenic patients, presenting with either generalized or focal abdominal pain, accompanied by fever, should be considered at risk of typhlitis [3]. The best imaging clue from a CT scan is a hypodense circumferential wall thickening of caecum and/or ascending colon (due oedema of the wall) frequently associated with pericolonic inflammation. Treatment including bowel rest, broad-spectrum antibiotics and haemodynamic support, especially if started early, should lead to a prompt remission of symptoms and survival of the patient.

## Figures and Tables

**Figure 1:**
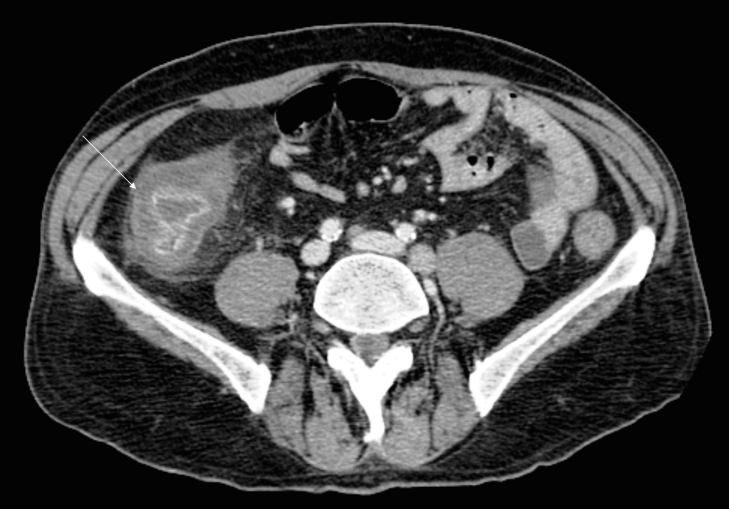
Post-contrast axial CT image, showing hypodense mural thickening of the caecum (arrow), with irregular contrast enhancement of the endoluminal surface.

**Figure 2:**
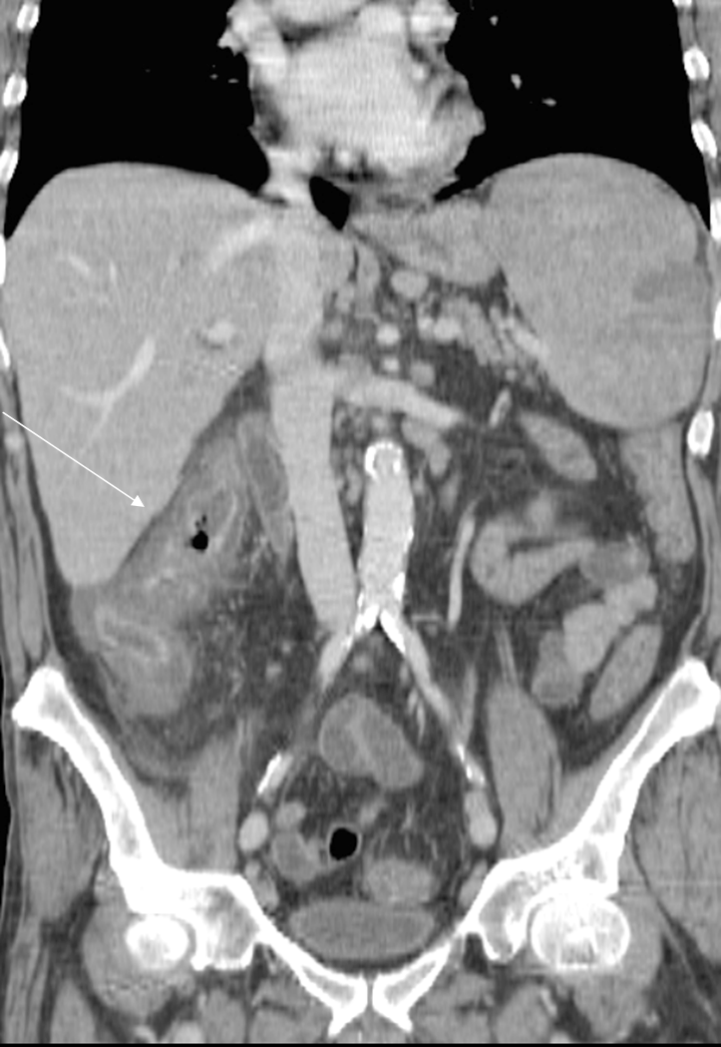
Post-contrast coronal reconstruction, showing the mural thickening extending from the caecum to the ascending colon (arrow).
